# Targeting Cellular and Tissue HIV Reservoirs With Toll-Like Receptor Agonists

**DOI:** 10.3389/fimmu.2019.02450

**Published:** 2019-10-15

**Authors:** Amanda B. Macedo, Camille L. Novis, Alberto Bosque

**Affiliations:** ^1^Department of Microbiology, Immunology and Tropical Medicine, George Washington University, Washington, DC, United States; ^2^Department of Pathology, Division of Microbiology and Immunology, The University of Utah, Salt Lake City, UT, United States

**Keywords:** HIV, toll-like receptors, latency-reversal agents, shock and kill, reservoirs

## Abstract

The elimination of both cellular and tissue latent reservoirs is a challenge toward a successful HIV cure. “Shock and Kill” are among the therapeutic strategies that have been more extensively studied to target these reservoirs. These strategies are aimed toward the reactivation of the latent reservoir using a latency-reversal agent (LRA) with the subsequent killing of the reactivated cell either by the cytotoxic arm of the immune system, including NK and CD8 T cells, or by viral cytopathic mechanisms. Numerous LRAs are currently being investigated *in vitro, ex vivo* as well as *in vivo* for their ability to reactivate and reduce latent reservoirs. Among those, several toll-like receptor (TLR) agonists have been shown to reactivate latent HIV. In humans, there are 10 TLRs that recognize different pathogen-associated molecular patterns. TLRs are present in several cell types, including CD4 T cells, the cell compartment that harbors the majority of the latent reservoir. Besides their ability to reactivate latent HIV, TLR agonists also increase immune activation and promote an antiviral response. These combined properties make TLR agonists unique among the different LRAs characterized to date. Additionally, some of these agonists have shown promise toward finding an HIV cure in animal models. When in combination with broadly neutralizing antibodies, TLR-7 agonists have shown to impact the SIV latent reservoir and delay viral rebound. Moreover, there are FDA-approved TLR agonists that are currently being investigated for cancer therapy and other diseases. All these has prompted clinical trials using TLR agonists either alone or in combination toward HIV eradication approaches. In this review, we provide an extensive characterization of the state-of-the-art of the use of TLR agonists toward HIV eradication strategies and the mechanism behind how TLR agonists target both cellular and tissue HIV reservoirs.

## Introduction

HIV infection is still one of the highest causes of mortality and morbidity worldwide. The introduction of anti-retroviral therapy (ART) in 1996 decreased the mortality due to HIV infection and transformed the disease from deadly to chronic. Cure is still not attainable due to a small reservoir of infected cells that harbor the virus in a latent form and become unrecognizable by the immune system and current therapies (1–5). Several strategies have been proposed to eliminate this latent reservoir (6). Among these strategies, the “shock and kill” approach rely on the notion that a pharmacological agent that reactivates the latent virus or latency-reversing agent (LRA) in the presence of ART will reduce the latent reservoir and could be followed by two potential outcomes. “Shock and kill” strategies can lead to complete viral eradication. In this case, the final goal is to eliminate all latent viruses. In a way, these strategies will try to echo the Berlin or the London patient, whom underwent remission due to bone marrow transplants and became undetectable for viral reservoirs (7, 8). Another outcome could be that a reduction of the latent reservoir due to the “shock and kill” strategy may be sufficient to allow the immune system control viral replication in the absence of ART (9–12). This has been termed “functional cure” and it is exemplified with the VISCONTI study. In this study, a subset of HIV-infected individuals who started ART early were able to control viremia in the absence of ART (termed Post-Treatment Controllers or PTCs) (11, 12). The authors of this study found a strong association between a low HIV reservoir in blood with the ability of the immune system to control viremia after ART treatment interruption (11).

As of today, several LRAs have been developed and some have reached clinical trials. The first generation LRAs that reached human testing include Valproic Acid (13–18), SAHA (19–21), Romidepsin (22), Panobinostat (23), Bryostatin-1 (24), and Disulfiram (25, 26). However, these LRAs have resulted in limited to no clinical effect on the size of the latent reservoir (27–29). Some potential explanations for the failure of these LRAs are the following. First, the lack of or low reactivation of latent viruses with these LRAs *in vivo*. Second, the reduced killing of reactivated cells either by the low frequency or compartmentalization of HIV-specific cells on patients under ART, immune exhaustion or the presence of defective proviruses that divert the immune response from the reactivated cells carrying replication competent virus. Third, a survival advantage of latently infected cells (30–36). To that end, strategies that can efficiently reactivate latent HIV *in vivo* and also enhance immune responses against HIV may overcome these obstacles encountered by the current cure efforts.

Recently, a second generation of LRAs targeting toll-like receptors (TLRs) have reached clinical trials. TLRs are pathogen-recognition receptors (PRRs) capable of sensing small molecular motifs conserved within microbes (37, 38). In addition to their ability to reactivate latent HIV, TLR agonists also increase immune activation and promote antiviral responses (39–44). These combined properties make TLR agonists unique among the LRAs characterized to date.

In 1891, William Coley demonstrated how several bacterial components could be used to treat cancer patients (45). Since then, several TLR ligands are being investigated and in clinical trials to enhance immunity for their use in treatment of cancer (46), viral infection (47), and bacterial infection (48). Several reviews have previously focused in the development and use of TLR agonists for cancer and other diseases (49–51). Here, we provide a comprehensive literature review specifically focused on the development of TLR agonists as LRAs and their potential use of these agonists for HIV eradication purposes.

## Toll-Like Receptors

### TLRs and Their Ligands

**Figure 1:**
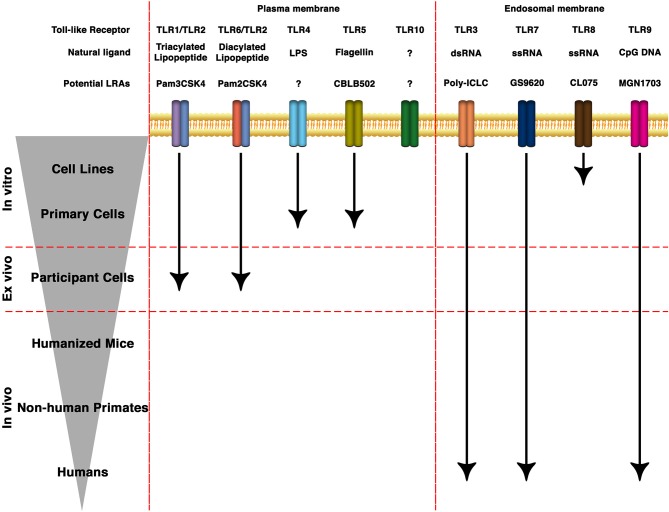
State of the art of TLRs as LRAs for HIV eradication. TLRs can be expressed either in the plasma membrane or in endosomal membranes. They recognize molecular patterns such as lipids, proteins, nucleic acids and carbohydrates present in bacteria, protozoa, viruses or fungi. Based on the structure of the natural ligands, several synthetic derivatives and small molecules have been developed to specifically target different TLRs. Several of them are being investigated toward HIV cure strategies in cell lines, animal models or clinical trials.

TLRs are transmembrane PRRs that recognize a plethora of molecules present in virus, bacteria, fungi or protozoa such as lipids, proteins, nucleic acids, and carbohydrates (52, 53). PRRs are germline-encoded receptors which function as first line of detection of pathogenic infections and recognize conserved molecular structures called pathogen-associated molecular patterns (PAMPs) (54). PRRs can also recognize soluble molecules released during cell death or damage. These structures are called damage-associated molecular patterns (DAMPs) (55, 56). In humans, there are 10 TLRs that differ both in their location within the cell as well as their cognate PAMP (Figure 1). TLR-1, 2, 4, 5, 6, and 10 are localized on the surface of the cells and recognize PAMPs present at the exterior of bacteria, fungi, and protozoa. On the other hand, TLR-3, 7, 8, and 9 are localized within endosomal structures and recognize nucleic acids derived from bacteria and viruses (53, 57). TLRs recognize their cognate ligand through either homodimers or heterodimers and are expressed in cells of the innate and adaptive immune system (such as dendritic cells, macrophages, granulocytes, T cells, B cells, NK cells, and mast cells) as well as epithelial and endothelial cells [reviewed in (37)].

#### TLRs in the Plasma Membrane

TLR-2 has a broader spectrum of ligand recognition than other TLRs due to its ability to dimerize with other receptors (58, 59). TLR-2 can recognize diacylated lipopeptides in the surface of gram-positive bacteria in conjunction with TLR-6 (60, 61). Whereas, TLR-2 can recognize triacylated lipopeptides present in gram-negative bacteria together with TLR-1 (62). TLR-2 has been shown to also induce signaling as a homodimer when recognizing lipoarabidomannan of *Mycobacterium smegmatis* (LAM-MS) and polysaccharide A of *Bacteroides fragilis* (PSA) (63, 64). Finally, TLR-2 can complex to the c-type lectin Dectin-1 to recognize zymosan, a β-glucan present in yeast cell wall (65). TLR-4 recognizes lipopolysaccharide (LPS), the principal component of gram-negative bacteria, and its truncated versions lipooligosaccharide and lipid A (66). CD14 and MD-2 are also needed for proper TLR-4 signaling (67, 68). TLR-5 recognizes flagellin, the main component of bacterial flagella (69). Lastly, TLR-10 has been an orphan receptor for a long time with no clearly defined ligand (70). TLR-10 has been shown to be involved in triacylated lipopeptides recognition by TLR-2 (71, 72). Recently, TLR-10 has been shown to sense gp41 and other HIV proteins in conjunction with TLR-1 and TLR-2 (73).

#### TLRs in the Endosomal Membrane

Several TLRs are present in endosomes, lysosomes and endolysosomes (74). TLR-3 recognizes double-stranded RNA (dsRNA) generated during viral infections while TLR-7 and TLR-8 both recognize single stranded RNA (ssRNA) (75, 76). Finally, TLR-9 can recognize unmethylated double-stranded DNA derived from both bacteria and viruses (77).

#### Synthetic Ligands

Due to their immunostimulatory properties, the discovery of small molecules that can “mimic” a TLR response is an area of active research. Over the past years, several small molecules have been developed to specifically target TLRs and are being investigated for the treatment of bacterial and viral infections, for cancer immunotherapy and to optimize vaccine efficacy [reviewed in (50, 78, 79)].

Synthetic lipopeptides have been the gold standard TLR-2 ligands. Pam2CSK4 is a synthetic diacylated lipopeptide that engages TLR-2/TLR-6 heterodimers or TLR-2 homodimers (60, 80). Pam3CSK4 is a synthetic triacylated lipopeptide that engages TLR-2/TLR-1 heterodimers (62, 81). Mono-acyl lipopeptides are the minimal structure required for TLR-2 activity (82). Guan and colleagues identified a series of compounds with a similar core structure consisting of 3-carboxylbenzothiophene linked via a carbonothioyl amino bridge to an anilino group (83). Further structure-activity relationship (SAR) studies have yielded an optimized novel compound termed CU-T12-9 (84). This compound has shown higher efficacy than the original compound and shows a specificity toward TLR-2/TLR-1 heterodimers over TLR-2/TLR-6 heterodimers (84). Using structure-based virtual screening of over 10.5 million compounds, Chen and colleagues identified ethyl 2-(4-methylpiperazine-1-carboxamido)-5,6-dihydro-4H-cyclopenta[b]thiophene-3-carboxylate (SMU127) as a specific TLR-2/TLR-1 heterodimer ligand (85). Recently, the same group has developed 2-(1-(2-(Methylamino)-5-nitrophenyl)-1H-imidazol-4-yl)-5-(trifluoromethyl)phenol (SMU-Z1) as a specific TLR-2/TLR-1 heterodimer ligand (86). Finally, a screening in PMA-differentiated THP-1 cell line of nearly 100,000 compounds identified diprovocims as inducers of TLR-2 and TLR-1 receptor dimerization and activation in the low pM range (87).

The main synthetic ligand used for TLR-3 is polyinosinic-polycytidylic acid [poly(I:C)]. Poly(I:C) mimics dsRNA and it is formed of a strand of inosine poly(I) homopolymer annealed to a strand of cytidine poly(C) homopolymer. The antiviral and antitumoral activities of Poly(I:C) were described in the 1960's but it was not characterized as a TLR-3 agonist until the early 2000's (75). Other derivatives of Poly(I:C) have been developed such as a combination of polyinosinic-polycytidylic acid and poly-L-lysine (Poly- ICLC/Hiltonol^®^), or the introduction of uridine in the Poly(I:C) strand (poly(I:C_12_U)/rintatolimod/Ampligen^®^), or Polyadenylic–polyuridylic acid [poly(A:U)] (88–90).

Monophosphoryl lipid A (MPL) is a detoxified form of the TLR4 agonist LPS from *Salmonella minnesota* that retains immunostimulatory properties but lacks the toxic effects of LPS (91). RC599 is a synthetic mimetic of MPL derived from aminoalkyl glucosaminide 4-phosphate (92). Both, MPL and RC599 have been shown to be efficient adjuvants and promote CD4 T cell responses (93). MPL has been used in a number of complex adjuvants included in human vaccines (Supervax^®^, Cervarix, Melacine^®^, Stimuvax) [reviewed in (94)]. Other lipid A mimetics (AS04, GLA-SE, GSK1795091, and OM-174) have been developed as vaccine adjuvants and are in licensed vaccines or in Phase I or II clinical trials as anticancer therapeutics [reviewed in (95)].

The polypeptide CBLB502 (Entolimod) derived from Flagellin is a potent TLR-5 ligand under extensive investigation as vaccine adjuvant, cancer and ischemia (96–99).

Imiquimod is an imidazoquinoline amine analog to guanosine that specifically activates TLR-7 (100). Several other agonists derived of imidazoquinoline have been developed that target TLR-7 such as Gardiquimod™ and PF-04878691 [also known as 852A or 3M-001) (101, 102)], or to target TLR-7 and TLR-8 simultaneously such as CL075, CL097, or Resiquimod (also known as R-848) (103–105). In addition, guanosine analogs such as Loxoribine have been generated as specific TLR-7 ligands (106). ANA975 and ANA773, also guanosine analogs, are prodrugs derived of the TLR-7 agonist isatoribine (107, 108). CL264 and SM360320 are derivatives of 9-benzyl-8 hydroxyadenine that have been shown to stimulate TLR-7 (109, 110). Finally, GS-9620 (Vesatolimod) and its close analog GS-986 are also a 9-benzyl-8 hydroxyadenine derivatives that targets TLR-7 with higher activity than CL264 (41, 42, 111).

All the synthetic TLR-9 ligands generated to date are derived from CpG oligodeoxynucleotides (CpG ODNs). CpGs ODNs can be classified into three classes (A, B, or C) that differ in their structure as well as their immunostimulatory properties (112, 113). Among others, CpG ODNs include CPG10101, IMO-2125, SD-101, CpG7909 (ProMune), MGN1703 (Lefitolimod) (114–118).

Finally, a series of synthetic TLR ligands that covalently link two or more TLR ligands are under investigation. These multi-TLR ligands have been designed to enhance immune responses through the synergistic activation of two or more TLRs with different downstream pathways (42, 119).

### TLR Signaling Pathway

**Figure 2:**
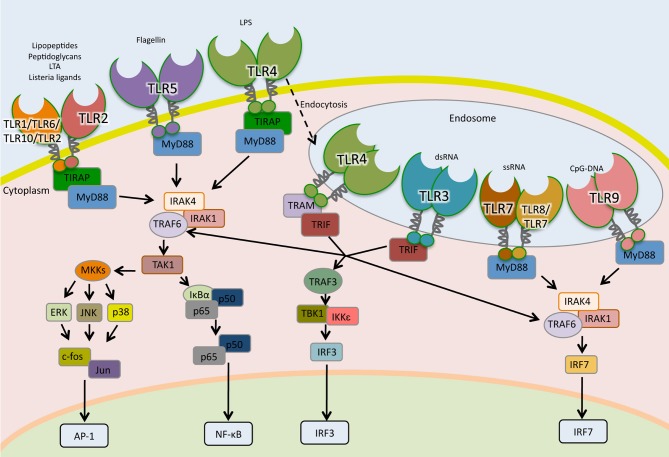
Toll-like receptors signaling pathways. TLRs are the sentinels of host defense. The homodimers TLR5, TLR4, and TLR2 and the heterodimers TLR2-TLR1, TLR2-TLR6, and TLR2-TLR10 bind to their specific ligand at the cell surface, whereas TLR3, TLR7, TLR7-TLR8, and TLR9 localize to the endosomes, where they interact to their ligands. TLR4 following microbial detection is endocytosed into the endosome. When TLRs are activated by interaction with their ligands, adaptor molecules are recruited to stimulate downstream signaling pathways including NF- κB, AP1, and IRFs.

TLRs are type I transmembrane glycoproteins composed of a leucine-rich-repeat (LRR) motifs on the extracellular or endosomal domain that mediates ligand recognition and the Toll/IL-1R (TIR) in the cytoplasmic domain responsible for signaling (120). The signaling cascades following TLR activation involves multiple steps. Adaptor proteins such as myeloid differentiation primary-response protein 88 (MyD88), TIR-domain containing adaptor protein (TIRAP, also known as MAL), TIR-domain containing adaptor protein inducing IFN-β (TRIF) and TRIF-related adaptor molecule (TRAM) are recruited to the TIR domain after interaction of PAMPs to their cognate TLR (Figure 2).

With the exception of TLR-3, all TLRs signal through the MyD88-dependent pathway. In the MyD88-dependent pathway, TLR-2 and TLR-4 require TIRAP in order to recruit MyD88 to start signal transduction while TLR-5, TLR-7, TLR-8, and TLR-9 initiate signaling using uniquely MyD88 (121, 122). After recruitment of MyD88, a complex is formed with interleukin-1 receptor-associated kinase-1 (IRAK1) and IRAK4. IRAK1 is phosphorylated and associates with TNF receptor-associated factor 6 (TRAF6), which activates transforming growth factor-β-activated kinase 1 (TAK1), also known as mitogen-activated protein kinase kinase kinase 7 (MAP3K7). TAK1 activates, by phosphorylation, two routes; the IκB kinase—nuclear factor kappa-light-chain-enhancer of activated B cells (IKK-NF-κB) pathway and the mitogen-activated protein kinases (MAPK) pathway (Figure 2). In the first route, TAK-1 phosphorylates I-kappa-B-α/β (IκBα/β). This phosphorylation leads to their degradation through the proteasome system and the release of NF-κB, which translocates to the nucleus, binds to DNA and initiates transcription. In the second route, TAK1 activates the MAPK members extracellular signal-regulated kinase (ERK), c-Jun N-terminal kinase (JNK) and p38, which lead to activation of the nuclear factor activator protein-1 (AP-1) (57, 123). In plasmocytoid dendritic cells (pDCs), TLR-7, and TLR-9 activate MyD88 signaling that leads to the phosphorylation and activation of the transcription factor interferon regulatory factor 7 (IRF7), which regulates the expression of IFN-α (124).

In the MyD88-independent pathway, TRIF is recruited to TLR-3 to initiate signaling (125). Besides TLR-3, TLR-4 can be endocytosed and signal through TRIF using the adaptor molecule TRAM (126, 127). TRAF6 and TRAF3 are recruited to TRIF. While TRAF6 engages IKK and MAPK, leading to the activation of NF-κB, AP-1, and IRF7; TRAF3 recruits TBK1/IKK-ε complex that activates IRF3 culminating in IFN-β expression (Figure 2) (128, 129).

## Toll-Like Receptor Agonists as Latency-Reversing Agents

### *In vitro* Studies

The importance of TLRs in the physiopathology of HIV was first postulated after observations on increased plasma viral loads seen in HIV-infected individuals exposed to vaccination regimens, suffering of opportunistic bacterial infections or sexually transmitted diseases, or had translocation of microbial products from the gut (130–138). Early studies have shown that PAMPs and their corresponding microorganisms transactivate the HIV long-terminal repeat (LTR) promoter. For example, it was described that purified protein derivative (PPD) of *Mycobacterium tuberculosis* increased viral mRNA expression in HIV infected monocytes (139). Furthermore, monocytic cell lines stimulated with live *M. tuberculosis* or lipomannan (LAM) increased p24 expression by 3-fold and enhanced HIV LTR transcription (140). Additionally, it has been shown that both *M. tuberculosis* PPD from the H37Ra strain and the mycobacterial major cell wall component mannosylated LAM (ManLAM) activated transcription of HIV in the T cell line Jurkat. ManLAM-induced HIV gene expression was mediated via protein kinases that culminated in NF-κB nuclear translocation. Mutations in the NF-κB binding sites in the HIV LTR abolished PDD-induced HIV expression (141, 142). These findings suggested that microbial products could be inducing HIV transcription. The latter discovery of TLRs as sensors of these microbial products suggested the idea that TLR agonists could reactivate latent HIV and could be potential LRAs.

Subsequently increasing number of reports have demonstrated the role of specific TLR agonists as latency reversing agents *in vitro*. Equils and colleagues transfected human dermal endothelial cells with an LTR luciferase construct and showed that stimulation of TLR-4 with LPS leads to NF-κB activation and transactivation of HIV-LTR (143). However, the effect of LPS in the reactivation of the HIV promoter in T cells has been disputed by other groups (144–146). These divergent results could be explained by the use of contaminated LPS formulations with other PAMPs, like bacterial lipopeptides. Other possible reasons for the discrepancy could be variation in the cell type used in each study, since different cells respond differently to TLR agonists, the cell culture environment, which component could escalate or impair an agonists effect, and/or disparities in TLR-4 binding affinity to LPS from different bacteria. Recently, LPS has been shown to reactivate latent HIV in macrophages isolated from the urethra of patients under ART (147).

The TLR-9 agonist CpG ODNs has been shown to activate HIV replication in the chronically infected human cell lines U1 and ACH2 in an NF-κB dependent manner (148, 149). Furthermore, DNA from *F. nucleatum* increased HIV promoter activity through TLR-9 signaling in the THP89GFP cell line (144). Recently, the TLR9 MGN1703 has been shown to induced HIV RNA release in peripheral blood mononuclear cells (PBMCs) from aviremic HIV-infected donors on antiretroviral therapy (ART) (150).

Mycobacteria have been shown to induce HIV transcription in a TLR-2 dependent manner. Bhat et al. observed that the *M. tuberculosis* and *M. smegmatis* proline-proline-glutamic acid protein Rv1168c (PPE17) interacts with TLR-2 resulting in activation of NF-κB and HIV transactivation in the human monocytic cell line THP1 (151). Our group has shown that the TLR-2 agonists Pam2CSK4 and Pam3CSK4 have latency reversal activity in CD4 T cells from aviremic HIV-infected participants and in a primary T_CM_ cell model of latency (42, 146, 152). Finally, the component of the *M. tuberculosis* membrane phosphatidylinositol mannoside 6 (PIM6) as well as whole bacteria in co-culture reactivated HIV in a primary T_CM_ cell model of latency. As for previous studies with *M. tuberculosis*, viral reactivation was dependent on TLR-2 (153).

R-848, a TLR-7/-8 agonist, induced p24 expression in the latently infected monocytic cell lines U1 and OM10 (154). Furthermore, a combination of the PKC agonist and LRA prostratin with a TLR-8 agonist 3M-002 was tested in a coculture of latently infected cells (J-Lat) and monocyte-derived dendritic cells (MDDCs). The combination of protratin and 3M-002 resulted in greater reactivation of HIV latency in J-Lat than each compounds alone. This synergistic interaction was dependent on TNF-α and on MDDC-T cell interactions (155).

Flagellin, the structural protein in bacterial flagella and a TLR5 agonist, has been shown to reactivate latent HIV in J-Lat, a transformed cell line latently infected with HIV derived of Jurkat (146, 156, 157). Thibault and colleagues also shown activity of flagellin in central memory T cells previously infected with a VSV-G pseudotyped NL43. However, resting CD4 T cells from aviremic patients, when challenged with flagellin, failed to elicit detectable levels of viral gene expression (157).

The TLR-3 agonist Poly(I:C) has been shown to reactivate latent HIV via NF-κB and JNK in the monocytic cell line U38 that contains a stably integrated and silent copy of the HIV LTR promoter linked to the chloramphenicol acetyltransferase (CAT) gene (158).

The selective TLR-7 agonist GS-9620 induced extracellular HIV RNA release in the supernatants of PBMCs isolated from HIV-infected participants on ART but not in purified CD4 T cells (159). In this study, Tsai and colleagues attributed this viral reactivation to type I IFN produced by pDCs. Therefore, this last finding suggests two complementary mechanisms of latency reversal mediated by TLR agonists. One that requires a subset of immune cells to be activated with the TLR agonist and these cells release soluble factors with latency reversal activity. And a second mechanism in which the TLR agonists have a direct effect on latently infected CD4T cells. Based on this idea, we have recently characterized the mechanisms of viral reactivation mediated by the TLR-2 agonist Pam2CSK4 and the TLR-7 agonist GS-9620 and compared with that of synthetic dual TLR-2 and TLR-7 agonists (dual TLR-2/7 agonists). We found that TLR-2 and TLR-7 agonists reactivate latency by two distinct and complementary mechanisms. TLR-2 agonists reactivate HIV by directly inducing NF-κB activation in memory CD4 T cells, while TLR7 agonists induced the secretion of soluble factors that can reactivate latent HIV in CD4 T cells. Our results suggests that TNF-α but not type-I IFN secreted by monocytes and plasmacytoid dendritic cells (pDCs) promotes viral reactivation in CD4 T cells (42).

TLR agonists have also shown to reactivate latent HIV in other cell subsets besides CD4 T cells and monocytes/macrophages. Poly (I:C) and bacterial ribosomal RNA induced HIV reactivation through TLR-3 in a latency model using immortalized human primary microglia with simian virus-40 (SV40) large T antigen and human telomerase reverse transcriptase (160). Furthermore, stimulation with TLR-2, TLR-4, or TLR-9 agonists induced HIV replication in a primary latency model using human progenitor mast cells (161).

### *In vivo* Studies: From Small Animals to Clinical Trials

Mouse models have been a tool to study the role of TLR agonists on the pathogenesis of HIV. Initial studies were done using a transgenic mouse model that contains intact copies of HIV proviral DNA (162). Infection of these animals with *Mycobacterium avium* or *Toxoplasma gondii* increased viral production in monocytes/macrophages (163, 164). Using *ex vivo* spleens from these transgenic mice, Equils and colleagues demonstrated that ligands for TLR-2 (soluble Mycobacterium tuberculosis factor or STF), TLR-4 (LPS), and TLR-9 (CpG) increased viral production and that combination of LPS with either STF or CpG increased viral production in an additive manner (165). Furthermore, Bafica and colleagues crossed this HIV transgenic mouse with either a TLR-2-deficient or a control mouse to investigate the role of TLR-2 in the activation of HIV expression. Culture filtrate proteins, phosphatidyl-inositol mannoside from *M. tuberculosis* and the synthetic lipopeptdide Pam3CSK4 induced p24 expression in spleen cells from HIV transgenic mouse expressing TLR-2 but not the TLR-2 deficient mice (166).

Several studies have specifically looked at the effects of TLR agonists on the latent reservoir *in vivo* (Table 1). A study using a humanized mice model of HIV latency demonstrated that the TLR-3 agonists poly(I:C) can reactivate latent HIV *in vivo* (167). In this study, the co-administration of poly(I:C) with a vaccination regimen including recombinant anti-human CD40 antibody fused to 5 HIV peptide regions (αCD40.HIV5pep) reduced the levels of cell-associated HIV DNA and delayed 1 week viral rebound after ART interruption in these animals (167). The selective TLR-7 small-molecule agonist GS-9620 has demonstrated antiviral activity in animal models of hepatitis B virus, good safety profiles and has progressed to clinical testing (171–173). As such, TLR-7 agonists have also been tested as LRAs in SIV-infected rhesus macaques. So far, there has been 4 different studies on the administration of GS-9620 or its analog GS-986 in infected macaques under suppressive ART. The first study combined the therapeutic vaccine Ad26/MVA (recombinant adenovirus serotype 26 (Ad26) prime, modified vaccinia Ankara (MVA) boost) with GS-986. They found that this combination resulted in decreased levels of viral DNA in lymph nodes and peripheral blood, and improved virologic control and delayed viral rebound following discontinuation of antiretroviral therapy (39). In another study from the same group, GS-9620 was combined with the V3 glycan-dependent bNAb PGT121 in rhesus monkeys during ART (40). This combination delayed viral rebound after ART discontinuation. Interestingly, 5 out of 11 monkeys did not show viral rebound even after CD8-depletion or adoptive transfer of PBMCs and LNMCs into naïve monkeys (40). In a third study, repeated administration of GS-9620 or its analog G-986 to SIVmac251-infected rhesus macaques under ART was associated with transient plasma viremia that peaked 24 to 48 h after dosing (41). Furthermore, TLR-7 agonists induced a reduction in cell-associated SIV DNA in sorted memory CD4 T cells obtained from peripheral blood, gastrointestinal mucosa and lymph nodes (41). Finally, a recent study also evaluated the effects of repeated doses of the TLR7 agonist GS-9620 in SIV-infected rhesus macaques receiving ART (169). The rhesus macaques that received GS-9620 during ART presented with immunologic effects due to the TLR-7 agonist, such as upregulation of IFN-stimulated genes in both blood and tissues, an increase in different plasma cytokines such as IFN-α and IL-1RA, and changes in CD8, NK and macrophage activation. In contrast with the study from Lim and colleagues, GS-9620 did not result in a measurable increase in plasma viremia or changes in viral RNA–to–viral DNA ratio in PBMCs or tissues, nor decreases in viral DNA in PBMC or tissues (169). Several differences can account for the discrepancies in these two studies. First, ART was initiated at different times after initial infection (65 vs. 13 days). Second, GS-9620 was administrated at different times post-ART initiation (60 vs. 75 weeks). Third, the SIV strain used in each study were different (SIVmac251 vs. SIVmac239X). Forth, the route of inoculation was also different (intrarectally vs. intravenously). All of these differences could lead to variations in the formation of viral reservoirs and their sensitivity to TLR-7 agonists. Further studies are warranted to fully understand the discrepancies of these two studies.

**Table 1:** Summary of *in vivo* studies using TLR ligands as LRAs.

	**Administration**	**Study arms**	**Specie**	**Viral blips**	**Effects on reservoir**	**References**
Poly I:C (TLR3)	Two doses 2.5 weeks apart, half IP half IM	PBS Poly I:C αCD40.HIV5pep/ Poly I:C	NRG hu-mice	Yes (US/7)	Reduction on cell-associated DNA in lymphoid tissue (4/4) Delayed rebound (αCD40.HIV5pep/Poly I:C 1 week)	(167)
Poly-ICLC (TLR3)	Two consecutively daily doses, SC	Randomize (3:1) vs placebo	Human	0/12	Reduction on cell-associated DNA (0/12)	(168)
GS-986 (TLR7)	Ten doses two weeks apart, OG	Sham Ad26/MVA GS-986 GS-986/Ad26/MVA	Rhesus macaque	Sham (0/9) Ad26/MVA (0/9) GS-986 (0/9) GS-986/Ad26/MVA (1/9)	Reduction on cell-associated DNA in lymph nodes week 70 (Sham 1/9, Ad26/MVA 1/9, GS-986 4/8; GS-986/Ad26/MVA 6/8) Reduction on cell-associated DNA in PBMCs week 70 (Sham 3/9, Ad26/MVA 9/9, GS-986 4/8; GS-986/Ad26/MVA 6/8) Virologic control (Sham 0/9, Ad26/MVA 0/9, GS-986 0/8, GS-986/Ad26/MVA 3/8)	(39)
GS-9620 (TLR7)	Ten doses two weeks apart, OG	Sham PGT121 GS-9620 GS-9620/PGT121	Rhesus macaque	Sham (0/11) PGT121 (0/11) GS-9620 (0/11) GS-9620/ PGT121 (0/11)	Reduction of viral DNA in lymph nodes week 120 (Sham 4/11, PGT121 7/11, GS-9620 3/11, GS-9620/PGT121 11/11) Virologic control (Sham 0/11, PGT121 3/11, GS-9620 1/11, GS-9620/PGT121 5/11)	(40)
GS-986 (TLR7)	Dose scalation 2 weeks apart, OG	Sham GS-986	Rhesus macaque	Sham (0/3) GS-986 (4/4)	Reduction of viral DNA in memory CD4 T cells from PBMCs (3/4), LNMCs (4/4) and GMMCs (4/4)	(41)
GS-9620 (TLR7)	Ten doses 2 weeks apart, OG	Sham GS-9620 0.05 mg/kg GS-9620 0.1 mg/kg GS-9620 0.15 mg/kg	Rhesus macaque	Sham (0/3) GS-9620 0.05 mg/kg (3/3) GS-9620 0.1 mg/kg (3/3) GS-9620 0.15 mg/kg (3/3)	Reduction of viral DNA in memory CD4 T cells from PBMCs (Sham 2/2, GS-9620 0.05 mg/kg 3/3, GS-9620 0.1 mg/kg 3/3, GS-9620 0.15 mg/kg 3/3) Reduction of viral DNA in memory CD4 T cells from LNMCs (Sham 1/2, GS-9620 0.05 mg/kg 1/3, GS-9620 0.1 mg/kg 2/3, GS-9620 0.15 mg/kg 1/3) Reduction of viral DNA in memory CD4 T cells from GMMCs (Sham 0/2, GS-9620 0.05 mg/kg 2/3, GS-9620 0.1 mg/kg 3/3, GS-9620 0.15 mg/kg 3/3) Virologic control (Sham 0/2, GS-9620 0.05 mg/kg 0/3, GS-9620 0.1 mg/kg 1/3, GS-9620 0.15 mg/kg 1/3)	(41)
GS-9620 (TLR7)	First course of twelve doses 2 weeks apart, OG Second course of five dose 2 weeks apart, OG	Sham GS-9620	Rhesus macaque	Sham (0/2) GS-9620 (0/4)	No effects in viral HIV DNA	(169)
GS-9620 (TLR7)	Tablet(s) administered orally once every 2 weeks	Randomized, blinded, placebo-controlled dose-escalation study	Human	Completed trial	Completed trial	NCT02858401
GS-9620 (TLR7)	Up to 10 doses administered as four 2 mg tablets orally every 14 days	Randomized, Double-Blind, Placebo-controlled Study	Human	Ongoing trial	Ongoing trial	NCT03060447
CpG-ODN 7909 (TLR9)	Immunized with double the standard dose of PCV7 (Prevnar^®^, Wyeth) at 0 and 3 months and with one single dose of PPV-23 (Pneumo Novum^®^, Sanofi-Pasteur MSD)	Placebo CpG-ODN 7909	Human	ND	Decrease in viral HIV DNA in PBMCs in CpG-ODN 7909 group relative to placebo control	(170)
MGN1703 (TLR9)	Eight doses twice weekly for 4 week, SC	Single-arm, open-label study	Human	6/15	No effects in viral HIV DNA in CD4 T cells	(43)
MGN1703 (TLR9)	Forty-eight doses twice weekly for 24 week, SC	Single-arm, open-label study	Human	ND	No effects in viral HIV DNA in CD4 T cells No changes in CA US HIV RNA in CD4 T cells No changes in IUPM	(44)

*IP, intraperitoneally; IM, intramuscularly; SC, subcutaneously; OG, oral gavage; NRG mice, NOD.Cg-Rag1^tm1Mom^Il2rg^tm1Wjl^/SzJ mice; US, Unspecified; ND, Non-determined; CA US, cell-associated unspliced; IUPM, infectious units per million*.

TLR agonists have also reached clinical testing in ART-suppressed participants (Table 1). The TLR-9 agonist CpG-ODN 7909, a class B CpG ODN, was administered as adjuvant in HIV-infected individuals (174). Interestingly, those participants receiving the TLR9 agonist as adjuvant in the immunization protocol had some decrease in the HIV proviral reservoir compared the control group (170). This led to the idea of using TLR-9 agonists to reduce the latent reservoir. As such, two different clinical trials aimed toward HIV eradication have been done using the TLR-9 ligand MGN1703. In the first clinical trial, a single-arm, open-label study in which 15 (13 male, 2 female) virologically suppressed HIV infected individuals on ART received 60 mg MGN1703 subcutaneously twice weekly for 4 weeks (43). In this study, they characterized pDC, NK, and T-cell activation using flow cytometry on baseline and after 4 weeks of treatment. Additionally, HIV transcription was quantified by measuring plasma HIV RNA. MGN1703 treatment increased the activation of pDCs, upregulated levels of cytokines, and enhanced activation of cytotoxic NK cells and effector CD8^+^ T cells. Furthermore, treatment with MGN1703 induced plasma HIV RNA blips up to >1,500 copies/mL in 6 of 15 participants (43). From this trial, biopsies from sigmoid colon were collected from 11 participants. Increased in solely type-1 interferon response but not a broad inflammatory response was observed in intestinal mononuclear cells. Interestingly, increased transcription of either *TLR9* or *IFNAR1* before MGN1703 administration was associated with improved efficacy in eliminating HIV DNA-containing cells in the intestine during the course of treatment (175). In the second clinical trial, the same group enrolled HIV-infected individuals on ART for an exploratory, single-arm clinical trial that tested the safety and immune enhancement effects of 24-weeks of MGN1703 (60 mg 2 weekly) therapy (44). A total of twelve individuals completed the treatment phase and nine underwent analytical treatment interruption (ATI). MGN1703 led to potent T-cell activation and increased HIV-specific T-cell responses, however there were no changes in CD4 T cells containing viral DNA nor differences in the time to rebound after ATI. In this study, a single patient was able to control viremia for 150 days after ATI. This participant had strong HIV-specific cellular and antibody-mediated immune responses, however as the study did not contain a placebo arm, the delayed viral rebound cannot exclusively be associated to MGN1703 treatment (44). The TLR-3 agonist Poly-ICLC (Hiltonol^®^) has also been tested in a randomized, placebo-controlled, double-blinded trial in ART-suppressed participants (168). In this study, participants received two daily doses of Poly-ICLC subcutaneously. Both, Poly-ICLC and placebo control were observed for adverse events, immune activation, and viral replication. As for other TLR agonists, Poly-ICLC administration lead to transient innate immune stimulation without generalized immune activation. While no effects of Poly-ICLC in reversing HIV latency or on the size of the viral reservoirs were observed; Poly-ICLC was reported safe and well-tolerated (168). Finally, the TLR7 agonist GS-9620 is currently being evaluated in clinical trials in HIV infected controllers (NCT03060447) and in those on suppressive ART (NCT02858401). These studies will provide information regarding safety and biological activity, including their impact on viral reservoirs, in HIV-infected patients.

## Modulation of HIV-host Immune Responses by TLR Stimulation

Besides their potential ability to reactivate latent HIV, TLR agonists have been shown to have immunostimulatory and antiviral properties to modulate anti-HIV immune responses.

The TLR-2 agonist Pam3CSK4 has been shown to prime latently-infected CD4 T cells for CD8 T cell recognition (176). We have characterized that TLR-2 and dual TLR-2/7 agonists, besides reactivating latent HIV, they can activate NK cells and induce IL-22. We demonstrated a protective role for IL-22 in both cell-free and cell-to-cell HIV infection of CD4 T cells (42). Whether TLR-2 agonists enhance the ability of NK cells to kill HIV-infected cells has not been demonstrated yet. Furthermore, the dual TLR-2/7 agonist PamadiFectin has been shown to enhance humoral responses in a mouse model immunized with p24 coupled to nanoparticles (119).

The TLR-3 agonist poly(I:C) enhanced the generation of HIV-specific T cell responses in BALB/c mice and in humanize mouse vaccination models (167, 177). *In vitro*, the TLR-7 agonist GS-9620 has been shown to both inhibit HIV replication in an IFN-α-mediated mechanism as well as enhance the anti-HIV activity of CD8 T and NK cells (159, 178). The TLR-7/8 agonist 3M-012, an analog of R-848, has been given as vaccine adjuvant in combination with HIV Gag antigen to non-human primates. The addition of 3M-012 to the Gag vaccine substantially enhanced Gag-specific T helper 1 and CD8 T cell responses compared to animals given the Gag protein alone (179). Furthermore, TLR-7/-8 activation through ssRNA or R-848 interfered with HIV replication cycle in lymphocyte cultures (154). In addition, The TLR-7/8 agonists 3M-002 and R-848 were able to promote HIV control *in vitro* in HIV-infected PBMCs through the activation of CD8 T cells and NK cells (180).

A randomized controlled vaccine trial conducted with 95 HIV-infected subjects investigated the impact of TLR-9 agonist as an adjuvant for pneumococcal vaccine. The trial showed that the TLR-9 agonist, CpG-ODN 7909, enhanced vaccine immunogenicity in the experimental group compared with the control group (174). *Post-hoc* analyses of the vaccine trial confirmed that patients that received TLR-9 ligand as adjuvant expressed more CD107a and macrophage inflammatory protein 1β (MIP1β) markers in CD8 T cells. The increase in these markers was associated with a reduction in HIV proviral load (170). Furthermore, the TLR-9 agonist CpG-ODN 2216 has been shown to activate NK cells in a pDC-mediated mechanism and enhance NK lysis of autologous HIV-infected CD4 T cells (181). In addition, TLR ligands such as Imiquimod (TLR-7), R-848 (TLR-7/8), CpG ODN (TLR-9), and Poly(I:C) (TLR-3) have been also shown to enhance the generation of HIV-specific CD8 T cells *in vitro* (182).

## Concluding Remarks

The use of TLR ligands as LRAs has shown promising results in efforts toward HIV eradication either alone or in combination with other therapeutic strategies because of their ability to reactivate latent HIV, to enhance immune responses and promote antiviral responses. Several TLR agonists are under investigation both in pre-clinical models of HIV latency as well as in clinical trials (Figure 1). These preclinical and clinical studies have shown a wide variety of response even in studies using the same TLR agonists (Table 1). Why such differences are currently unknown. Based on these studies, we can speculate several factors that may be influencing the response to TLR agonists including the time of ART initiation, the length of ART treatment, the cellular composition of the latent reservoir, or the strain of SIV or HIV. There are also other factors that may influence the activity of these agonists that will need to be considered in future cure strategies. First, TLRs contain polymorphisms that influence their activity (183, 184). How these polymorphisms affect the efficacy of different TLR ligands in HIV eradication approaches has not been characterized. Second, it is well-known that biological sex influences the responses of certain TLRs, in particular TLR-7 (185, 186). The *in vivo* evaluation of GS-9620 or other TLR-7 agonists will need to take this into account. As research progresses, emphasis also needs to be done in understanding whether these TLR agonists can reach all the different tissue compartments where HIV may hide, including lymph nodes, intestinal mucosal, and brain [reviewed in (187–191)]. Furthermore, HIV has been shown to reside latent in other cell types besides CD4 T cell (147, 192). As such, it is possible that a single TLR agonist may not be sufficient to reactivate all latent virus present in different cellular compartments as the expression of TLRs differs among different cell subsets. Additional research is warranted to fully understand which TLR agonists reactivate latent HIV in each cell compartment. Also, it will be important to address whether reactivation is due to a direct targeting of the TLR in the reservoir cell or whether other soluble factors secreted by other cells are required for efficient viral reactivation. The mechanisms involved in HIV latency are complex and involve a plethora of cellular factors as well as epigenetic mechanisms (193). As such, TLR agonists may need to be combined with other LRAs with different mechanisms of action to efficiently reactivate all latent viruses. Finally, recent studies using animal models suggest the use of additional strategies, such as bNAbs, might be required to generate meaningful remission. Therefore, it is important to continue the investigation of TLR agonists as potential adjuvants for novel HIV cure strategies.

## Author Contributions

AM, CN, and AB contributed to the conception of the review and wrote the first draft of the manuscript. All authors contributed to manuscript revision, read, and approved the submitted version.

### Conflict of Interest

The authors declare that the research was conducted in the absence of any commercial or financial relationships that could be construed as a potential conflict of interest.

## References

[1] Chun TW Stuyver L Mizell SB Ehler LA Mican JA Baseler M. Presence of an inducible HIV-1 latent reservoir during highly active antiretroviral therapy. Proc Natl Acad Sci USA. (1997) 94:13193–7. 10.1073/pnas.94.24.13193 9371822 PMC24285

[2] Finzi D Hermankova M Pierson T Carruth LM Buck C Chaisson RE. Identification of a reservoir for HIV-1 in patients on highly active antiretroviral therapy. Science. (1997) 278:1295–300. 10.1126/science.278.5341.1295 9360927

[3] Wong JK Hezareh M Gunthard HF Havlir DV Ignacio CC Spina CA. Recovery of replication-competent HIV despite prolonged suppression of plasma viremia. Science. (1997) 278:1291–5. 10.1126/science.278.5341.1291 9360926

[4] Brenchley JM Hill BJ Ambrozak DR Price DA Guenaga FJ Casazza JP. T-cell subsets that harbor human immunodeficiency virus (HIV) *in vivo*: implications for HIV pathogenesis. J Virol. (2004) 78:1160–8. 10.1128/JVI.78.3.1160-1168.2004 14722271 PMC321406

[5] Chomont N El-Far M Ancuta P Trautmann L Procopio FA Yassine-Diab B. HIV reservoir size and persistence are driven by T cell survival and homeostatic proliferation. Nat Med. (2009) 15:893–900. 10.1038/nm.1972 19543283 PMC2859814

[6] Barouch DH Deeks SG. Immunologic strategies for HIV-1 remission and eradication. Science. (2014) 345:169–74. 10.1126/science.1255512 25013067 PMC4096716

[7] Gupta RK Abdul-Jawad S McCoy LE Mok HP Peppa D Salgado M. HIV-1 remission following CCR5Delta32/Delta32 haematopoietic stem-cell transplantation. Nature. (2019) 568:244–8. 10.1038/s41586-019-1027-4 30836379 PMC7275870

[8] Hutter G Nowak D Mossner M Ganepola S Mussig A Allers K. Long-term control of HIV by CCR5 Delta32/Delta32 stem-cell transplantation. N Engl J Med. (2009) 360:692–8. 10.1056/NEJMoa0802905 19213682

[9] Cheret A Bacchus-Souffan C Avettand-Fenoel V Melard A Nembot G Blanc C. Combined ART started during acute HIV infection protects central memory CD4+ T cells and can induce remission. J Antimicrob Chemother. (2015) 70:2108–20. 10.1093/jac/dkv084 25900157

[10] Hocqueloux L Prazuck T Avettand-Fenoel V Lafeuillade A Cardon B Viard JP. Long-term immunovirologic control following antiretroviral therapy interruption in patients treated at the time of primary HIV-1 infection. AIDS. (2010) 24:1598–601. 10.1097/QAD.0b013e32833b61ba 20549847

[11] Saez-Cirion A Bacchus C Hocqueloux L Avettand-Fenoel V Girault I Lecuroux C. Post-treatment HIV-1 controllers with a long-term virological remission after the interruption of early initiated antiretroviral therapy ANRS VISCONTI Study. PLoS Pathog. (2013) 9:e1003211. 10.1371/journal.ppat.1003211 23516360 PMC3597518

[12] Samri A Bacchus-Souffan C Hocqueloux L Avettand-Fenoel V Descours B Theodorou I. Polyfunctional HIV-specific T cells in Post-Treatment Controllers. AIDS. (2016) 30:2299–302. 10.1097/QAD.0000000000001195 27428742

[13] Archin NM Cheema M Parker D Wiegand A Bosch RJ Coffin JM Antiretroviral intensification and valproic acid lack sustained effect on residual HIV-1 viremia or resting CD4+ cell infection. PLoS ONE. (2010) 5:e9390 10.1371/journal.pone.0009390 20186346 PMC2826423

[14] Archin NM Eron JJ Palmer S Hartmann-Duff A Martinson JA Wiegand A. Valproic acid without intensified antiviral therapy has limited impact on persistent HIV infection of resting CD4+ T cells. AIDS. (2008) 22:1131–5. 10.1097/QAD.0b013e3282fd6df4 18525258 PMC3863687

[15] Lehrman G Hogue IB Palmer S Jennings C Spina CA Wiegand A. Depletion of latent HIV-1 infection *in vivo*: a proof-of-concept study. Lancet. (2005) 366:549–55. 10.1016/S0140-6736(05)67098-5 16099290 PMC1894952

[16] Routy JP Tremblay CL Angel JB Trottier B Rouleau D Baril JG. Valproic acid in association with highly active antiretroviral therapy for reducing systemic HIV-1 reservoirs: results from a multicentre randomized clinical study. HIV Med. (2012) 13:291–6. 10.1111/j.1468-1293.2011.00975.x 22276680

[17] Sagot-Lerolle N Lamine A Chaix ML Boufassa F Aboulker JP Costagliola D Prolonged valproic acid treatment does not reduce the size of latent HIV reservoir. AIDS. (2008) 22:1125–9. 10.1097/QAD.0b013e3282fd6ddc 18525257

[18] Siliciano JD Lai J Callender M Pitt E Zhang H Margolick JB. Stability of the latent reservoir for HIV-1 in patients receiving valproic acid. J Infect Dis. (2007) 195:833–6. 10.1086/511823 17299713

[19] Archin NM Bateson R Tripathy MK Crooks AM Yang KH Dahl NP. HIV-1 expression within resting CD4+ T cells after multiple doses of vorinostat. J Infect Dis. (2014) 210:728–35. 10.1093/infdis/jiu155 24620025 PMC4148603

[20] Archin NM Liberty AL Kashuba AD Choudhary SK Kuruc JD Crooks AM. Administration of vorinostat disrupts HIV-1 latency in patients on antiretroviral therapy. Nature. (2012) 487:482–5. 10.1038/nature11286 22837004 PMC3704185

[21] Elliott JH Wightman F Solomon A Ghneim K Ahlers J Cameron MJ. Activation of HIV transcription with short-course vorinostat in HIV-infected patients on suppressive antiretroviral therapy. PLoS Pathog. (2014) 10:e1004473. 10.1371/journal.ppat.1004473 25393648 PMC4231123

[22] Sogaard OS Graversen ME Leth S Olesen R Brinkmann CR Nissen SK. The depsipeptide romidepsin reverses HIV-1 latency *in vivo*. PLoS Pathog. (2015) 11:e1005142. 10.1371/journal.ppat.1005142 26379282 PMC4575032

[23] Rasmussen TA Tolstrup M Brinkmann CR Olesen R Erikstrup C Solomon A. Panobinostat, a histone deacetylase inhibitor, for latent-virus reactivation in HIV-infected patients on suppressive antiretroviral therapy: a phase 1/2, single group, clinical trial. Lancet HIV. (2014) 1:e13–21. 10.1016/S2352-3018(14)70014-1 26423811

[24] Gutierrez C Serrano-Villar S Madrid-Elena N Perez-Elias MJ Martin ME Barbas C. Bryostatin-1 for latent virus reactivation in HIV-infected patients on antiretroviral therapy. AIDS. (2016) 30:1385–92. 10.1097/QAD.0000000000001064 26891037

[25] Elliott JH McMahon JH Chang CC Lee SA Hartogensis W Bumpus N. Short-term administration of disulfiram for reversal of latent HIV infection: a phase 2 dose-escalation study. Lancet HIV. (2015) 2:e520–529. 10.1016/S2352-3018(15)00226-X 26614966 PMC5108570

[26] Spivak AM Andrade A Eisele E Hoh R Bacchetti P Bumpus NN. A pilot study assessing the safety and latency-reversing activity of disulfiram in HIV-1-infected adults on antiretroviral therapy. Clin Infect Dis. (2014) 58:883–90. 10.1093/cid/cit813 24336828 PMC3935499

[27] Rasmussen TA Schmeltz Sogaard O Brinkmann C Wightman F Lewin SR Melchjorsen J. Comparison of HDAC inhibitors in clinical development: effect on HIV production in latently infected cells and T-cell activation. Hum Vaccin Immunother. (2013) 9:993–1001. 10.4161/hv.23800 23370291 PMC3899169

[28] Spivak AM Planelles V. HIV-1 eradication: early trials (and Tribulations). Trends Mol Med. (2016) 22:10–27. 10.1016/j.molmed.2015.11.004 26691297 PMC5889129

[29] Van Lint C Bouchat S Marcello A. HIV-1 transcription and latency: an update. Retrovirology. (2013) 10:67. 10.1186/1742-4690-10-67 23803414 PMC3699421

[30] Day CL Kaufmann DE Kiepiela P Brown JA Moodley ES Reddy S. PD-1 expression on HIV-specific T cells is associated with T-cell exhaustion and disease progression. Nature. (2006) 443:350–4. 10.1038/nature05115 16921384

[31] Ho YC Shan L Hosmane NN Wang J Laskey SB Rosenbloom DI. Replication-competent noninduced proviruses in the latent reservoir increase barrier to HIV-1 cure. Cell. (2013) 155:540–51. 10.1016/j.cell.2013.09.020 24243014 PMC3896327

[32] Huang SH Ren Y Thomas AS Chan D Mueller S Ward AR. Latent HIV reservoirs exhibit inherent resistance to elimination by CD8+ T cells. J Clin Invest. (2018) 128:876–89. 10.1172/JCI97555 29355843 PMC5785246

[33] Kuo HH Ahmad R Lee GQ Gao C Chen HR Ouyang Z. Anti-apoptotic Protein BIRC5 Maintains Survival of HIV-1-Infected CD4(+) T Cells. Immunity. (2018) 48:1183–94.e1185. 10.1016/j.immuni.2018.04.004 29802019 PMC6013384

[34] Pollack RA Jones RB Pertea M Bruner KM Martin AR Thomas AS. Defective HIV-1 proviruses are expressed and can be recognized by cytotoxic T lymphocytes, which shape the proviral landscape. Cell Host Microbe. (2017) 21:494–506.e494. 10.1016/j.chom.2017.03.008 28407485 PMC5433942

[35] Shan L Deng K Shroff NS Durand CM Rabi SA Yang HC. Stimulation of HIV-1-specific cytolytic T lymphocytes facilitates elimination of latent viral reservoir after virus reactivation. Immunity. (2012) 36:491–501. 10.1016/j.immuni.2012.01.014 22406268 PMC3501645

[36] Trautmann L Janbazian L Chomont N Said EA Gimmig S Bessette B. Upregulation of PD-1 expression on HIV-specific CD8+ T cells leads to reversible immune dysfunction. Nat Med. (2006) 12:1198–202. 10.1038/nm1482 16917489

[37] Hopkins PA Sriskandan S. Mammalian Toll-like receptors: to immunity and beyond. Clin Exp Immunol. (2005) 140:395–407. 10.1111/j.1365-2249.2005.02801.x 15932500 PMC1809390

[38] Janeway CA Jr Medzhitov R. Innate immune recognition. Annu Rev Immunol. (2002) 20:197–216. 10.1146/annurev.immunol.20.083001.084359 11861602

[39] Borducchi EN Cabral C Stephenson KE Liu J Abbink P Ng'ang'a D. Ad26/MVA therapeutic vaccination with TLR7 stimulation in SIV-infected rhesus monkeys. Nature. (2016) 540:284–7. 10.1038/nature20583 27841870 PMC5145754

[40] Borducchi EN Liu J Nkolola JP Cadena AM Yu WH Fischinger S. Antibody and TLR7 agonist delay viral rebound in SHIV-infected monkeys. Nature. (2018) 563:360–4. 10.1038/s41586-018-0600-6 30283138 PMC6237629

[41] Lim SY Osuna CE Hraber PT Hesselgesser J Gerold JM Barnes TL. TLR7 agonists induce transient viremia and reduce the viral reservoir in SIV-infected rhesus macaques on antiretroviral therapy. Sci Transl Med. (2018) 10:aao4521. 10.1126/scitranslmed.aao4521 29720451 PMC5973480

[42] Macedo AB Novis CL De Assis CM Sorensen ES Moszczynski P Huang SH. Dual TLR2 and TLR7 agonists as HIV latency-reversing agents. JCI Insight. (2018) 3:e122673. 10.1172/jci.insight.122673 30282829 PMC6237480

[43] Vibholm L Schleimann MH Hojen JF Benfield T Offersen R Rasmussen K. Short-course toll-like receptor 9 agonist treatment impacts innate immunity and plasma viremia in individuals with human immunodeficiency virus infection. Clin Infect Dis. (2017) 64:1686–95. 10.1093/cid/cix201 28329286 PMC5849129

[44] Vibholm LK Konrad CV Schleimann MH Frattari G Winckelmann A Klastrup V Effects of 24 Week toll-like receptor 9 agonist treatment in HIV-1+ individuals: a single-arm, phase 1B/2A trial. AIDS. (2019) 33:1315–25. 10.1097/QAD.0000000000002213 30932955

[45] Coley WB. II. Contribution to the Knowledge of Sarcoma. Ann Surg. (1891) 14:199–220. 10.1097/00000658-189112000-00015 17859590 PMC1428624

[46] Vacchelli E Eggermont A Sautes-Fridman C Galon J Zitvogel L Kroemer G. Trial Watch: toll-like receptor agonists for cancer therapy. Oncoimmunology. (2013) 2:e25238. 10.4161/onci.25238 24083080 PMC3782517

[47] Horscroft NJ Pryde DC Bright H. Antiviral applications of Toll-like receptor agonists. J Antimicrob Chemother. (2012) 67:789–801. 10.1093/jac/dkr588 22258929

[48] Hancock RE Nijnik A Philpott DJ. Modulating immunity as a therapy for bacterial infections. Nat Rev Microbiol. (2012) 10:243–54. 10.1038/nrmicro2745 22421877

[49] Galluzzi L Vacchelli E Eggermont A Fridman WH Galon J Sautes-Fridman C. Trial Watch: experimental Toll-like receptor agonists for cancer therapy. Oncoimmunology. (2012) 1:699–716. 10.4161/onci.20696 22934262 PMC3429574

[50] Savva A Roger T. Targeting toll-like receptors: promising therapeutic strategies for the management of sepsis-associated pathology and infectious diseases. Front Immunol. (2013) 4:387. 10.3389/fimmu.2013.00387 24302927 PMC3831162

[51] Vacchelli E Galluzzi L Eggermont A Fridman WH Galon J Sautes-Fridman C. Trial watch: FDA-approved Toll-like receptor agonists for cancer therapy. Oncoimmunology. (2012) 1:894–907. 10.4161/onci.20931 23162757 PMC3489745

[52] Kawai T Akira S. The role of pattern-recognition receptors in innate immunity: update on Toll-like receptors. Nat Immunol. (2010) 11:373–84. 10.1038/ni.1863 20404851

[53] Kumar H Kawai T Akira S. Pathogen recognition by the innate immune system. Int Rev Immunol. (2011) 30:16–34. 10.3109/08830185.2010.529976 21235323

[54] Schnare M Holt AC Takeda K Akira S Medzhitov R. Recognition of CpG DNA is mediated by signaling pathways dependent on the adaptor protein MyD88. Curr Biol. (2000) 10:1139–42. 10.1016/S0960-9822(00)00700-4 10996797

[55] Bianchi ME. DAMPs, PAMPs and alarmins: all we need to know about danger. J Leukoc Biol. (2007) 81:1–5. 10.1189/jlb.0306164 17032697

[56] Venereau E Ceriotti C Bianchi ME. DAMPs from cell death to new life. Front Immunol. (2015) 6:422. 10.3389/fimmu.2015.00422 26347745 PMC4539554

[57] Akira S Uematsu S Takeuchi O. Pathogen recognition and innate immunity. Cell. (2006) 124:783–801. 10.1016/j.cell.2006.02.015 16497588

[58] Akira S Takeda K Toll-like receptor signalling. Nat Rev. (2004) 4:499–511. 10.1038/nri1391 15229469

[59] Ozinsky A Underhill DM Fontenot JD Hajjar AM Smith KD Wilson CB. The repertoire for pattern recognition of pathogens by the innate immune system is defined by cooperation between toll-like receptors. Proc Natl Acad Sci USA. (2000) 97:13766–71. 10.1073/pnas.250476497 11095740 PMC17650

[60] Kang JY Nan X Jin MS Youn SJ Ryu YH Mah S. Recognition of lipopeptide patterns by Toll-like receptor 2-Toll-like receptor 6 heterodimer. Immunity. (2009) 31:873–84. 10.1016/j.immuni.2009.09.018 19931471

[61] Takeuchi O Kawai T Muhlradt PF Morr M Radolf JD Zychlinsky A. Discrimination of bacterial lipoproteins by Toll-like receptor 6. Int Immunol. (2001) 13:933–40. 10.1093/intimm/13.7.933 11431423

[62] Jin MS Kim SE Heo JY Lee ME Kim HM Paik SG. Crystal structure of the TLR1-TLR2 heterodimer induced by binding of a tri-acylated lipopeptide. Cell. (2007) 130:1071–82. 10.1016/j.cell.2007.09.008 17889651

[63] Means TK Lien E Yoshimura A Wang S Golenbock DT Fenton MJ. The CD14 ligands lipoarabinomannan and lipopolysaccharide differ in their requirement for Toll-like receptors. J Immunol. (1999) 163:6748–55. 10586073

[64] Round JL Lee SM Li J Tran G Jabri B Chatila TA. The Toll-like receptor 2 pathway establishes colonization by a commensal of the human microbiota. Science. (2011) 332:974–7. 10.1126/science.1206095 21512004 PMC3164325

[65] Gantner BN Simmons RM Canavera SJ Akira S Underhill DM. Collaborative induction of inflammatory responses by dectin-1 and Toll-like receptor 2. J Exp Med. (2003) 197:1107–17. 10.1084/jem.20021787 12719479 PMC2193968

[66] Poltorak A He X Smirnova I Liu MY Van Huffel C Du X. Defective LPS signaling in C3H/HeJ and C57BL/10ScCr mice: mutations in Tlr4 gene. Science. (1998) 282:2085–8. 10.1126/science.282.5396.2085 9851930

[67] Chow JC Young DW Golenbock DT Christ WJ Gusovsky F. Toll-like receptor-4 mediates lipopolysaccharide-induced signal transduction. J Biol Chem. (1999) 274:10689–92. 10.1074/jbc.274.16.10689 10196138

[68] Shimazu R Akashi S Ogata H Nagai Y Fukudome K Miyake K. MD-2, a molecule that confers lipopolysaccharide responsiveness on Toll-like receptor 4. J Exp Med. (1999) 189:1777–82. 10.1084/jem.189.11.1777 10359581 PMC2193086

[69] Hayashi F Smith KD Ozinsky A Hawn TR Yi EC Goodlett DR. The innate immune response to bacterial flagellin is mediated by Toll-like receptor 5. Nature. (2001) 410:1099–103. 10.1038/35074106 11323673

[70] Chuang T Ulevitch RJ. Identification of hTLR10: a novel human Toll-like receptor preferentially expressed in immune cells. Biochim Biophys Acta. (2001) 1518:157–61. 10.1016/S0167-4781(00)00289-X 11267672

[71] Guan Y Ranoa DR Jiang S Mutha SK Li X Baudry J Human TLRs 10 and 1 share common mechanisms of innate immune sensing but not signaling. J Immunol. (2010) 184:5094–103. 10.4049/jimmunol.0901888 20348427

[72] Oosting M Cheng SC Bolscher JM Vestering-Stenger R Plantinga TS Verschueren IC. Human TLR10 is an anti-inflammatory pattern-recognition receptor. Proc Natl Acad Sci USA. (2014) 111:E4478–84. 10.1073/pnas.1410293111 25288745 PMC4210319

[73] Henrick BM Yao XD Zahoor MA Abimiku A Osawe S Rosenthal KL. TLR10 senses HIV-1 proteins and significantly enhances HIV-1 infection. Front Immunol. (2019) 10:482. 10.3389/fimmu.2019.00482 30930906 PMC6430187

[74] Akira S Takeda K Kaisho T. Toll-like receptors: critical proteins linking innate and acquired immunity. Nat Immunol. (2001) 2:675–80. 10.1038/90609 11477402

[75] Alexopoulou L Holt AC Medzhitov R Flavell RA. Recognition of double-stranded RNA and activation of NF-kappaB by Toll-like receptor 3. Nature. (2001) 413:732–8. 10.1038/35099560 11607032

[76] Heil F Hemmi H Hochrein H Ampenberger F Kirschning C Akira S. Species-specific recognition of single-stranded RNA via toll-like receptor 7 and 8. Science. (2004) 303:1526–9. 10.1126/science.1093620 14976262

[77] Hemmi H Takeuchi O Kawai T Kaisho T Sato S Sanjo H. A Toll-like receptor recognizes bacterial DNA. Nature. (2000) 408:740–5. 10.1038/35047123 11130078

[78] Ho NI Huis In 't Veld LGM Raaijmakers TK Adema GJ. Adjuvants enhancing cross-presentation by dendritic cells: the key to more effective vaccines? Front Immunol. (2018) 9:2874. 10.3389/fimmu.2018.02874 30619259 PMC6300500

[79] Smith M Garcia-Martinez E Pitter MR Fucikova J Spisek R Zitvogel L. Trial Watch: toll-like receptor agonists in cancer immunotherapy. Oncoimmunology. (2018) 7:e1526250. 10.1080/2162402X.2018.1526250 30524908 PMC6279325

[80] Buwitt-Beckmann U Heine H Wiesmuller KH Jung G Brock R Akira S. Toll-like receptor 6-independent signaling by diacylated lipopeptides. Eur J Immunol. (2005) 35:282–9. 10.1002/eji.200424955 15580661

[81] Redecke V Hacker H Datta SK Fermin A Pitha PM Broide DH. Cutting edge: activation of Toll-like receptor 2 induces a Th2 immune response and promotes experimental asthma. J Immunol. (2004) 172:2739–43. 10.4049/jimmunol.172.5.2739 14978071

[82] Agnihotri G Crall BM Lewis TC Day TP Balakrishna R Warshakoon HJ. Structure-activity relationships in toll-like receptor 2-agonists leading to simplified monoacyl lipopeptides. J Med Chem. (2011) 54:8148–60. 10.1021/jm201071e 22007676 PMC3228886

[83] Guan Y Omueti-Ayoade K Mutha SK Hergenrother PJ Tapping RI. Identification of novel synthetic toll-like receptor 2 agonists by high throughput screening. J Biol Chem. (2010) 285:23755–62. 10.1074/jbc.M110.116046 20504771 PMC2911294

[84] Cheng K Gao M Godfroy JI Brown PN Kastelowitz N Yin H. Specific activation of the TLR1-TLR2 heterodimer by small-molecule agonists. Sci Adv. (2015) 1:e1400139. 10.1126/sciadv.1400139 26101787 PMC4474499

[85] Chen Z Cen X Yang J Tang X Cui K Cheng K. Structure-based discovery of a specific TLR1-TLR2 small molecule agonist from the ZINC drug library database. Chem Commun. (2018) 54:11411–4. 10.1039/C8CC06618C 30246199

[86] Cen X Zhu G Yang J Yang J Guo J Jin J. TLR1/2 specific small-molecule agonist suppresses leukemia cancer cell growth by stimulating cytotoxic T lymphocytes. Adv Sci. (2019) 6:1802042. 10.1002/advs.201802042 31131189 PMC6523386

[87] Morin MD Wang Y Jones BT Mifune Y Su L Shi H. Diprovocims: a new and exceptionally potent class of toll-like receptor agonists. J Am Chem Soc. (2018) 140:14440–54. 10.1021/jacs.8b09223 30272974 PMC6209530

[88] Lepe-Zuniga JL Rotbein J Gutterman JU. Production of interferon-alpha induced by dsRNA in human peripheral blood mononuclear cell cultures: role of priming by dsRNA-induced interferons-gamma and -beta. J Interferon Res. (1989) 9:445–56. 10.1089/jir.1989.9.445 2502585

[89] Wang L Smith D Bot S Dellamary L Bloom A Bot A. Noncoding RNA danger motifs bridge innate and adaptive immunity and are potent adjuvants for vaccination. J Clin Invest. (2002) 110:1175–84. 10.1172/JCI0215536 12393853 PMC150792

[90] Zhu X Nishimura F Sasaki K Fujita M Dusak JE Eguchi J. Toll like receptor-3 ligand poly-ICLC promotes the efficacy of peripheral vaccinations with tumor antigen-derived peptide epitopes in murine CNS tumor models. J Transl Med. (2007) 5:10. 10.1186/1479-5876-5-10 17295916 PMC1802742

[91] Ulrich JT Myers KR. Monophosphoryl lipid A as an adjuvant. Past experiences and new directions. Pharm Biotechnol. (1995) 6:495–524. 10.1007/978-1-4615-1823-5_21 7551233

[92] Johnson DA Sowell CG Johnson CL Livesay MT Keegan DS Rhodes MJ. Synthesis and biological evaluation of a new class of vaccine adjuvants: aminoalkyl glucosaminide 4-phosphates (AGPs). Bioorg Med Chem Lett. (1999) 9:2273–8. 10.1016/S0960-894X(99)00374-1 10465560

[93] Thompson BS Chilton PM Ward JR Evans JT Mitchell TC. The low-toxicity versions of LPS, MPL adjuvant and RC529, are efficient adjuvants for CD4+ T cells. J Leukoc Biol. (2005) 78:1273–80. 10.1189/jlb.0305172 16204643

[94] Toussi DN Massari P. Immune adjuvant effect of molecularly-defined toll-like receptor ligands. Vaccines. (2014) 2:323–53. 10.3390/vaccines2020323 26344622 PMC4494261

[95] Zaffaroni L Peri F. Recent advances on Toll-like receptor 4 modulation: new therapeutic perspectives. Future Med Chem. (2018) 10:461–76. 10.4155/fmc-2017-0172 29380635

[96] Burdelya LG Krivokrysenko VI Tallant TC Strom E Gleiberman AS Gupta D. An agonist of toll-like receptor 5 has radioprotective activity in mouse and primate models. Science. (2008) 320:226–30. 10.1126/science.1154986 18403709 PMC4322935

[97] Fukuzawa N Petro M Baldwin WM III Gudkov AV Fairchild RL. A TLR5 agonist inhibits acute renal ischemic failure. J Immunol. (2011) 187:3831–9. 10.4049/jimmunol.1003238 21890657 PMC3178726

[98] Mizel SB Bates JT. Flagellin as an adjuvant: cellular mechanisms and potential. J Immunol. (2010) 185:5677–82. 10.4049/jimmunol.1002156 21048152 PMC3756556

[99] Yoon SI Kurnasov O Natarajan V Hong M Gudkov AV Osterman AL. Structural basis of TLR5-flagellin recognition and signaling. Science. (2012) 335:859–64. 10.1126/science.1215584 22344444 PMC3406927

[100] Lee J Chuang TH Redecke V She L Pitha PM Carson DA. Molecular basis for the immunostimulatory activity of guanine nucleoside analogs: activation of Toll-like receptor 7. Proc Natl Acad Sci USA. (2003) 100:6646–51. 10.1073/pnas.0631696100 12738885 PMC164501

[101] Gorden KB Gorski KS Gibson SJ Kedl RM Kieper WC Qiu X. Synthetic TLR agonists reveal functional differences between human TLR7 and TLR8. J Immunol. (2005) 174:1259–68. 10.4049/jimmunol.174.3.1259 15661881

[102] Kalali BN Kollisch G Mages J Muller T Bauer S Wagner H. Double-stranded RNA induces an antiviral defense status in epidermal keratinocytes through TLR3-, PKR-, and MDA5/RIG-I-mediated differential signaling. J Immunol. (2008) 181:2694–704. 10.4049/jimmunol.181.4.2694 18684960

[103] Ahonen CL Gibson SJ Smith RM Pederson LK Lindh JM Tomai MA. Dendritic cell maturation and subsequent enhanced T-cell stimulation induced with the novel synthetic immune response modifier R-848. Cell Immunol. (1999) 197:62–72. 10.1006/cimm.1999.1555 10555997

[104] Petricevic B Wessner B Sachet M Vrbanec D Spittler A Bergmann M. CL097, a TLR7/8 ligand, inhibits TLR-4–dependent activation of IRAK-M and BCL-3 expression. Shock. (2009) 32:484–90. 10.1097/SHK.0b013e3181a5ac8a 19333135

[105] Spranger S Javorovic M Burdek M Wilde S Mosetter B Tippmer S. Generation of Th1-polarizing dendritic cells using the TLR7/8 agonist CL075. J Immunol. (2010) 185:738–47. 10.4049/jimmunol.1000060 20511554

[106] Pope BL Chourmouzis E Sigindere J Capetola RJ Lau CY. *in vivo* enhancement of murine natural killer cell activity by 7-allyl-8-oxoguanosine (loxoribine). Int J Immunopharmacol. (1992) 14:1375–82. 10.1016/0192-0561(92)90008-9 1464469

[107] Bergmann JF de Bruijne J Hotho DM de Knegt RJ Boonstra A Weegink CJ. Randomised clinical trial: anti-viral activity of ANA773, an oral inducer of endogenous interferons acting via TLR7, in chronic HCV. Aliment Pharmacol Ther. (2011) 34:443–53. 10.1111/j.1365-2036.2011.04745.x 21707679

[108] Xiang AX Webber SE Kerr BM Rueden EJ Lennox JR Haley GJ. Discovery of ANA975: an oral prodrug of the TLR-7 agonist isatoribine. Nucleosides Nucleotides Nucleic Acids. (2007) 26:635–40. 10.1080/15257770701490472 18066870

[109] Hayashi T Gray CS Chan M Tawatao RI Ronacher L McGargill MA. Prevention of autoimmune disease by induction of tolerance to Toll-like receptor 7. Proc Natl Acad Sci USA. (2009) 106:2764–9. 10.1073/pnas.0813037106 19188597 PMC2634806

[110] Hilbert T Steinhagen F Weisheit C Baumgarten G Hoeft A Klaschik S. Synergistic stimulation with different TLR7 ligands modulates gene expression patterns in the human plasmacytoid dendritic cell line CAL-1. Mediators Inflamm. (2015) 2015:948540. 10.1155/2015/948540 26770023 PMC4684865

[111] Rebbapragada I Birkus G Perry J Xing W Kwon H Pflanz S. Molecular determinants of GS-9620-dependent TLR7 activation. PLoS ONE. (2016) 11:e0146835. 10.1371/journal.pone.0146835 26784926 PMC4718629

[112] Liu Y Luo X Yang C Yu S Xu H. Three CpG oligodeoxynucleotide classes differentially enhance antigen-specific humoral and cellular immune responses in mice. Vaccine. (2011) 29:5778–84. 10.1016/j.vaccine.2011.05.087 21664398

[113] Vollmer J Weeratna R Payette P Jurk M Schetter C Laucht M. Characterization of three CpG oligodeoxynucleotide classes with distinct immunostimulatory activities. Eur J Immunol. (2004) 34:251–62. 10.1002/eji.200324032 14971051

[114] Agrawal S Kandimalla ER. Synthetic agonists of Toll-like receptors 7, 8 and 9. Biochem Soc Trans. (2007) 35:1461–7. 10.1042/BST0351461 18031246

[115] Paul S. Technology evaluation: CpG-7909, Coley. Curr Opin Mol Ther. (2003) 5:553–9. 14601526

[116] Vicari AP Schmalbach T Lekstrom-Himes J Morris ML Al-Adhami MJ Laframboise C. Safety, pharmacokinetics and immune effects in normal volunteers of CPG 10101 (ACTILON), an investigational synthetic toll-like receptor 9 agonist. Antivir Ther. (2007) 12:741–51. 17713157

[117] Wang S Campos J Gallotta M Gong M Crain C Naik E. Intratumoral injection of a CpG oligonucleotide reverts resistance to PD-1 blockade by expanding multifunctional CD8+ T cells. Proc Natl Acad Sc USA. (2016) 113:E7240–9. 10.1073/pnas.1608555113 27799536 PMC5135381

[118] Wittig B Schmidt M Scheithauer W Schmoll HJ. MGN1703, an immunomodulator and toll-like receptor 9 (TLR-9) agonist: from bench to bedside. Crit Rev Oncol Hematol. (2015) 94:31–44. 10.1016/j.critrevonc.2014.12.002 25577571

[119] Gutjahr A Papagno L Nicoli F Lamoureux A Vernejoul F Lioux T. Cutting edge: a Dual TLR2 and TLR7 ligand induces highly potent humoral and cell-mediated immune responses. J Immunol. (2017) 198:4205–9. 10.4049/jimmunol.1602131 28432147

[120] Yamamoto M Takeda K Akira S. TIR domain-containing adaptors define the specificity of TLR signaling. Mol Immunol. (2004) 40:861–8. 10.1016/j.molimm.2003.10.006 14698224

[121] Verstak B Nagpal K Bottomley SP Golenbock DT Hertzog PJ Mansell A. MyD88 adapter-like (Mal)/TIRAP interaction with TRAF6 is critical for TLR2- and TLR4-mediated NF-kappaB proinflammatory responses. J Biol Chem. (2009) 284:24192–203. 10.1074/jbc.M109.023044 19592497 PMC2782013

[122] Yamamoto M Sato S Hemmi H Sanjo H Uematsu S Kaisho T. Essential role for TIRAP in activation of the signalling cascade shared by TLR2 and TLR4. Nature. (2002) 420:324–9. 10.1038/nature01182 12447441

[123] Kawasaki T Kawai T. Toll-like receptor signaling pathways. Front Immunol. (2014) 5:461. 10.3389/fimmu.2014.00461 25309543 PMC4174766

[124] Ning S Pagano JS Barber GN. IRF7: activation, regulation, modification and function. Genes Immun. (2011) 12:399–414. 10.1038/gene.2011.21 21490621 PMC4437765

[125] Yamamoto M Sato S Hemmi H Hoshino K Kaisho T Sanjo H. Role of adaptor TRIF in the MyD88-independent toll-like receptor signaling pathway. Science. (2003) 301:640–3. 10.1126/science.1087262 12855817

[126] Kagan JC Su T Horng T Chow A Akira S Medzhitov R. TRAM couples endocytosis of Toll-like receptor 4 to the induction of interferon-beta. Nat Immunol. (2008) 9:361–8. 10.1038/ni1569 18297073 PMC4112825

[127] Yamamoto M Sato S Hemmi H Uematsu S Hoshino K Kaisho T. TRAM is specifically involved in the Toll-like receptor 4-mediated MyD88-independent signaling pathway. Nat Immunol. (2003) 4:1144–50. 10.1038/ni986 14556004

[128] Hacker H Redecke V Blagoev B Kratchmarova I Hsu LC Wang GG. Specificity in Toll-like receptor signalling through distinct effector functions of TRAF3 and TRAF6. Nature. (2006) 439:204–7. 10.1038/nature04369 16306937

[129] Xie P. TRAF molecules in cell signaling and in human diseases. J Mol Signal. (2013) 8:7. 10.1186/1750-2187-8-7 23758787 PMC3697994

[130] Bentwich Z Maartens G Torten D Lal AA Lal RB. Concurrent infections and HIV pathogenesis. AIDS. (2000) 14:2071–81. 10.1097/00002030-200009290-00002 11061647

[131] Brichacek B Swindells S Janoff EN Pirruccello S Stevenson M. Increased plasma human immunodeficiency virus type 1 burden following antigenic challenge with pneumococcal vaccine. J Infect Dis. (1996) 174:1191–9. 10.1093/infdis/174.6.1191 8940208

[132] Donovan RM Bush CE Markowitz NP Baxa DM Saravolatz LD. Changes in virus load markers during AIDS-associated opportunistic diseases in human immunodeficiency virus-infected persons. J Infect Dis. (1996) 174:401–3. 10.1093/infdis/174.2.401 8699074

[133] Goletti D Weissman D Jackson RW Graham NM Vlahov D Klein RS. Effect of Mycobacterium tuberculosis on HIV replication. Role of immune activation. J Immunol. (1996) 157:1271–8. 8757635

[134] Mole L Ripich S Margolis D Holodniy M. The impact of active herpes simplex virus infection on human immunodeficiency virus load. J Infect Dis. (1997) 176:766–70. 10.1086/517297 9291329

[135] O'Brien WA Grovit-Ferbas K Namazi A Ovcak-Derzic S Wang HJ Park J. Human immunodeficiency virus-type 1 replication can be increased in peripheral blood of seropositive patients after influenza vaccination. Blood. (1995) 86:1082–9. 7620162

[136] Stanley SK Ostrowski MA Justement JS Gantt K Hedayati S Mannix M. Effect of immunization with a common recall antigen on viral expression in patients infected with human immunodeficiency virus type 1. N Engl J Med. (1996) 334:1222–30. 10.1056/NEJM199605093341903 8606717

[137] Staprans SI Hamilton BL Follansbee SE Elbeik T Barbosa P Grant RM. Activation of virus replication after vaccination of HIV-1-infected individuals. J Exp Med. (1995) 182:1727–37. 10.1084/jem.182.6.1727 7500017 PMC2192265

[138] Sulkowski MS Chaisson RE Karp CL Moore RD Margolick JB Quinn TC. The effect of acute infectious illnesses on plasma human immunodeficiency virus (HIV) type 1 load and the expression of serologic markers of immune activation among HIV-infected adults. J Infect Dis. (1998) 178:1642–8. 10.1086/314491 9815216

[139] Toossi Z Xia L Wu M Salvekar A. Transcriptional activation of HIV by Mycobacterium tuberculosis in human monocytes. Clin Exp Immunol. (1999) 117:324–30. 10.1046/j.1365-2249.1999.00952.x 10444265 PMC1905327

[140] Zhang Y Nakata K Weiden M Rom WN. Mycobacterium tuberculosis enhances human immunodeficiency virus-1 replication by transcriptional activation at the long terminal repeat. J Clin Invest. (1995) 95:2324–31. 10.1172/JCI117924 7738195 PMC295846

[141] Bernier R Barbeau B Olivier M Tremblay MJ. Mycobacterium tuberculosis mannose-capped lipoarabinomannan can induce NF-kappaB-dependent activation of human immunodeficiency virus type 1 long terminal repeat in T cells. J Gen Virol. (1998) 79:1353–61. 10.1099/0022-1317-79-6-1353 9634075

[142] Lederman MM Georges DL Kusner DJ Mudido P Giam CZ Toossi Z. Mycobacterium tuberculosis and its purified protein derivative activate expression of the human immunodeficiency virus. J Acquir Immune Defic Syndr. (1994) 7:727–33. 8207650

[143] Equils O Faure E Thomas L Bulut Y Trushin S Arditi M. Bacterial lipopolysaccharide activates HIV long terminal repeat through Toll-like receptor 4. J Immunol. (2001) 166:2342–7. 10.4049/jimmunol.166.4.2342 11160291

[144] Gonzalez OA Li M Ebersole JL Huang CB. HIV-1 reactivation induced by the periodontal pathogens *Fusobacterium nucleatum* and *Porphyromonas gingivalis* involves Toll-like receptor 2 [corrected] and 9 activation in monocytes/macrophages. Clin Vaccine Immunol. (2010) 17:1417–27. 10.1128/CVI.00009-10 20610663 PMC2944464

[145] Nordone SK Ignacio GA Su L Sempowski GD Golenbock DT Li L. Failure of TLR4-driven NF-kappa B activation to stimulate virus replication in models of HIV type 1 activation. AIDS Res Hum Retroviruses. (2007) 23:1387–95. 10.1089/aid.2007.0033 18184082 PMC4415363

[146] Novis CL Archin NM Buzon MJ Verdin E Round JL Lichterfeld M. Reactivation of latent HIV-1 in central memory CD4(+) T cells through TLR-1/2 stimulation. Retrovirology. (2013) 10:119. 10.1186/1742-4690-10-119 24156240 PMC3826617

[147] Ganor Y Real F Sennepin A Dutertre CA Prevedel L Xu L. HIV-1 reservoirs in urethral macrophages of patients under suppressive antiretroviral therapy. Nat Microbiol. (2019) 4:633–44. 10.1038/s41564-018-0335-z 30718846

[148] Scheller C Ullrich A Lamla S Dittmer U Rethwilm A Koutsilieri E. Dual activity of phosphorothioate CpG oligodeoxynucleotides on HIV: reactivation of latent provirus and inhibition of productive infection in human T cells. Ann N Y Acad Sci. (2006) 1091:540–7. 10.1196/annals.1378.095 17341643

[149] Scheller C Ullrich A McPherson K Hefele B Knoferle J Lamla S. CpG oligodeoxynucleotides activate HIV replication in latently infected human T cells. J Biol Chem. (2004) 279:21897–902. 10.1074/jbc.M311609200 15016800

[150] Offersen R Nissen SK Rasmussen TA Ostergaard L Denton PW Sogaard OS. A novel toll-like receptor 9 agonist, MGN1703, enhances HIV-1 transcription and NK cell-mediated inhibition of HIV-1-infected autologous CD4+ T cells. J Virol. (2016) 90:4441–53. 10.1128/JVI.00222-16 26889036 PMC4836316

[151] Bhat KH Chaitanya CK Parveen N Varman R Ghosh S Mukhopadhyay S. Proline-proline-glutamic acid (PPE) protein Rv1168c of Mycobacterium tuberculosis augments transcription from HIV-1 long terminal repeat promoter. J Biol Chem. (2012) 287:16930–46. 10.1074/jbc.M111.327825 22427668 PMC3351301

[152] Macedo AB Resop RS Martins LJ Szaniawski MA Sorensen ES Spivak AM. Influence of biological sex, age and HIV status in an *in vitro* primary cell model of HIV latency using a CXCR4 tropic virus. AIDS Res Hum Retroviruses. (2018) 34:769–77. 10.1089/aid.2018.0098 29926732 PMC6152854

[153] Larson EC Novis CL Martins LJ Macedo AB Kimball KE Bosque A. *Mycobacterium tuberculosis* reactivates latent HIV-1 in T cells *in vitro*. PLoS ONE. (2017) 12:e0185162. 10.1371/journal.pone.0185162 28949981 PMC5614573

[154] Schlaepfer E Audige A Joller H Speck RF. TLR7/8 triggering exerts opposing effects in acute versus latent HIV infection. J Immunol. (2006) 176:2888–95. 10.4049/jimmunol.176.5.2888 16493046

[155] Rochat MA Schlaepfer E Speck RF. Promising role of toll-like receptor 8 agonist in concert with prostratin for activation of silent HIV. J Virol. (2017) 91:e02084–16. 10.1128/JVI.02084-16 27928016 PMC5286885

[156] Jordan A Bisgrove D Verdin E. HIV reproducibly establishes a latent infection after acute infection of T cells *in vitro*. EMBO J. (2003) 22:1868–77. 10.1093/emboj/cdg188 12682019 PMC154479

[157] Thibault S Imbeault M Tardif MR Tremblay MJ. TLR5 stimulation is sufficient to trigger reactivation of latent HIV-1 provirus in T lymphoid cells and activate virus gene expression in central memory CD4+ T cells. Virology. (2009) 389:20–5. 10.1016/j.virol.2009.04.019 19447460

[158] Bhargavan B Woollard SM Kanmogne GD. Toll-like receptor-3 mediates HIV-1 transactivation via NFkappaB and JNK pathways and histone acetylation, but prolonged activation suppresses Tat and HIV-1 replication. Cell Signal. (2016) 28:7–22. 10.1016/j.cellsig.2015.11.005 26569339 PMC4890564

[159] Tsai A Irrinki A Kaur J Cihlar T Kukolj G Sloan DD. Toll-like receptor 7 agonist GS-9620 induces HIV expression and HIV-specific immunity in cells from HIV-infected individuals on suppressive antiretroviral therapy. J Virol. (2017) 91:e02166–16. 10.1128/JVI.02166-16 28179531 PMC5375698

[160] Alvarez-Carbonell D Garcia-Mesa Y Milne S Das B Dobrowolski C Rojas R. Toll-like receptor 3 activation selectively reverses HIV latency in microglial cells. Retrovirology. (2017) 14:9. 10.1186/s12977-017-0335-8 28166799 PMC5294768

[161] Sundstrom JB Little DM Villinger F Ellis JE Ansari AA. Signaling through Toll-like receptors triggers HIV-1 replication in latently infected mast cells. J Immunol. (2004) 172:4391–401. 10.4049/jimmunol.172.7.4391 15034054

[162] Leonard JM Abramczuk JW Pezen DS Rutledge R Belcher JH Hakim F. Development of disease and virus recovery in transgenic mice containing HIV proviral DNA. Science. (1988) 242:1665–70. 10.1126/science.3201255 3201255

[163] Doherty TM Chougnet C Schito M Patterson BK Fox C Shearer GM. Infection of HIV-1 transgenic mice with Mycobacterium avium induces the expression of infectious virus selectively from a Mac-1-positive host cell population. J Immunol. (1999) 163:1506–15. 10415053

[164] Gazzinelli RT Sher A Cheever A Gerstberger S Martin MA Dickie P. Infection of human immunodeficiency virus 1 transgenic mice with *Toxoplasma gondii* stimulates proviral transcription in macrophages *in vivo*. J Exp Med. (1996) 183:1645–55. 10.1084/jem.183.4.1645 8666922 PMC2192489

[165] Equils O Schito ML Karahashi H Madak Z Yarali A Michelsen KS. Toll-like receptor 2 (TLR2) and TLR9 signaling results in HIV-long terminal repeat trans-activation and HIV replication in HIV-1 transgenic mouse spleen cells: implications of simultaneous activation of TLRs on HIV replication. J Immunol. (2003) 170:5159–64. 10.4049/jimmunol.170.10.5159 12734363

[166] Bafica A Scanga CA Schito ML Hieny S Sher A. Cutting edge: *in vivo* induction of integrated HIV-1 expression by mycobacteria is critically dependent on Toll-like receptor 2. J Immunol. (2003) 171:1123–7. 10.4049/jimmunol.171.3.1123 12874196

[167] Cheng L Wang Q Li G Banga R Ma J Yu H. TLR3 agonist and CD40-targeting vaccination induces immune responses and reduces HIV-1 reservoirs. J Clin Invest. (2018) 128:4387–96. 10.1172/JCI99005 30148455 PMC6159955

[168] Saxena M Sabado RL La Mar M Mohri H Salazar AM Dong H. Poly-ICLC, a TLR3 agonist, induces transient innate immune responses in patients with treated HIV-infection: a randomized double-blinded placebo controlled trial. Front Immunol. (2019) 10:725. 10.3389/fimmu.2019.00725 31024557 PMC6467168

[169] Del Prete GQ Alvord WG Li Y Deleage C Nag M Oswald K TLR7 agonist administration to SIV-infected macaques receiving early initiated cART does not induce plasma viremia. JCI Insight. (2019) 4:127717 10.1172/jci.insight.127717 31167974 PMC6629134

[170] Winckelmann AA Munk-Petersen LV Rasmussen TA Melchjorsen J Hjelholt TJ Montefiori D. Administration of a Toll-like receptor 9 agonist decreases the proviral reservoir in virologically suppressed HIV-infected patients. PLoS ONE. (2013) 8:e62074. 10.1371/journal.pone.0062074 23637967 PMC3637371

[171] Lanford RE Guerra B Chavez D Giavedoni L Hodara VL Brasky KM. GS-9620, an oral agonist of Toll-like receptor-7, induces prolonged suppression of hepatitis B virus in chronically infected chimpanzees. Gastroenterology. (2013) 144:1508–17, 1517.e1501–10. 10.1053/j.gastro.2013.02.003 23415804 PMC3691056

[172] Lopatin U Wolfgang G Tumas D Frey CR Ohmstede C Hesselgesser J. Safety, pharmacokinetics and pharmacodynamics of GS-9620, an oral Toll-like receptor 7 agonist. Antivir Ther. (2013) 18:409–18. 10.3851/IMP2548 23416308

[173] Menne S Tumas DB Liu KH Thampi L AlDeghaither D Baldwin BH. Sustained efficacy and seroconversion with the Toll-like receptor 7 agonist GS-9620 in the Woodchuck model of chronic hepatitis B. J Hepatol. (2015) 62:1237–45. 10.1016/j.jhep.2014.12.026 25559326 PMC4439359

[174] Sogaard OS Schonheyder HC Bukh AR Harboe ZB Rasmussen TA Ostergaard L. Pneumococcal conjugate vaccination in persons with HIV: the effect of highly active antiretroviral therapy. AIDS. (2010) 24:1315–22. 10.1097/QAD.0b013e328339fe0b 20559037

[175] Krarup AR Abdel-Mohsen M Schleimann MH Vibholm L Engen PA Dige A. The TLR9 agonist MGN1703 triggers a potent type I interferon response in the sigmoid colon. Mucosal Immunol. (2018) 11:449–61. 10.1038/mi.2017.59 28766555 PMC5796873

[176] Jones RB Mueller S O'Connor R Rimpel K Sloan DD Karel D. A subset of latency-reversing agents expose HIV-infected resting CD4+ T-cells to recognition by cytotoxic T-lymphocytes. PLoS Pathog. (2016) 12:e1005545. 10.1371/journal.ppat.1005545 27082643 PMC4833318

[177] Fujimoto C Nakagawa Y Ohara K Takahashi H. Polyriboinosinic polyribocytidylic acid [poly(I:C)]/TLR3 signaling allows class I processing of exogenous protein and induction of HIV-specific CD8+ cytotoxic T lymphocytes. Int Immunol. (2004) 16:55–63. 10.1093/intimm/dxh025 14688061

[178] Bam RA Hansen D Irrinki A Mulato A Jones GS Hesselgesser J. TLR7 agonist GS-9620 is a potent inhibitor of acute HIV-1 infection in human peripheral blood mononuclear cells. Antimicrob Agents Chemother. (2017) 61:e01369–16. 10.1128/AAC.01369-16 27799218 PMC5192112

[179] Wille-Reece U Wu CY Flynn BJ Kedl RM Seder RA. Immunization with HIV-1 Gag protein conjugated to a TLR7/8 agonist results in the generation of HIV-1 Gag-specific Th1 and CD8+ T cell responses. J Immunol. (2005) 174:7676–83. 10.4049/jimmunol.174.12.7676 15944268

[180] Schlaepfer E Speck RF. Anti-HIV activity mediated by natural killer and CD8+ cells after toll-like receptor 7/8 triggering. PLoS ONE. (2008) 3:e1999. 10.1371/journal.pone.0001999 18431484 PMC2292240

[181] Tomescu C Chehimi J Maino VC Montaner LJ. NK cell lysis of HIV-1-infected autologous CD4 primary T cells: requirement for IFN-mediated NK activation by plasmacytoid dendritic cells. J Immunol. (2007) 179:2097–104. 10.4049/jimmunol.179.4.2097 17675468

[182] Lore K Betts MR Brenchley JM Kuruppu J Khojasteh S Perfetto S. Toll-like receptor ligands modulate dendritic cells to augment cytomegalovirus- and HIV-1-specific T cell responses. J Immunol. (2003) 171:4320–8. 10.4049/jimmunol.171.8.4320 14530357

[183] Mukherjee S Huda S Sinha Babu SP. Toll-like receptor polymorphism in host immune response to infectious diseases: a review. Scand J Immunol. (2019) 90:e12771. 10.1111/sji.12771 31054156

[184] Skevaki C Pararas M Kostelidou K Tsakris A Routsias JG. Single nucleotide polymorphisms of Toll-like receptors and susceptibility to infectious diseases. Clin Exp Immunol. (2015) 180:165–77. 10.1111/cei.12578 25560985 PMC4408151

[185] Laffont S Rouquie N Azar P Seillet C Plumas J Aspord C. X-Chromosome complement and estrogen receptor signaling independently contribute to the enhanced TLR7-mediated IFN-alpha production of plasmacytoid dendritic cells from women. J Immunol. (2014) 193:5444–52. 10.4049/jimmunol.1303400 25339659

[186] Meier A Chang JJ Chan ES Pollard RB Sidhu HK Kulkarni S. Sex differences in the Toll-like receptor-mediated response of plasmacytoid dendritic cells to HIV-1. Nat Med. (2009) 15:955–9. 10.1038/nm.2004 19597505 PMC2821111

[187] Belmonte L Olmos M Fanin A Parodi C Bare P Concetti H. The intestinal mucosa as a reservoir of HIV-1 infection after successful HAART. AIDS. (2007) 21:2106–8. 10.1097/QAD.0b013e3282efb74b 17885303

[188] Lamers SL Rose R Maidji E Agsalda-Garcia M Nolan DJ Fogel GB. HIV DNA is frequently present within pathologic tissues evaluated at autopsy from combined antiretroviral therapy-treated patients with undetectable viral loads. J Virol. (2016) 90:8968–83. 10.1128/JVI.00674-16 27466426 PMC5044815

[189] Licht A Alter G. A Drug-Free Zone–Lymph Nodes as a Safe Haven for HIV. Cell Host Microbe. (2016) 19:275–6. 10.1016/j.chom.2016.02.018 26962938

[190] Rose R Nolan DJ Maidji E Stoddart CA Singer EJ Lamers SL. Eradication of HIV from tissue reservoirs: challenges for the cure. AIDS Res Hum Retroviruses. (2018) 34:3–8. 10.1089/aid.2017.0072 28691499 PMC5771544

[191] Yukl SA Shergill AK Ho T Killian M Girling V Epling L. The distribution of HIV DNA and RNA in cell subsets differs in gut and blood of HIV-positive patients on ART: implications for viral persistence. J Infect Dis. (2013) 208:1212–20. 10.1093/infdis/jit308 23852128 PMC3778964

[192] McNamara LA Ganesh JA Collins KL. Latent HIV-1 infection occurs in multiple subsets of hematopoietic progenitor cells and is reversed by NF-kappaB activation. J Virol. (2012) 86:9337–50. 10.1128/JVI.00895-12 22718820 PMC3416176

[193] Khoury G Darcis G Lee MY Bouchat S Van Driessche B Purcell DFJ. The molecular biology of HIV latency. Adv Exp Med Biol. (2018) 1075:187–212. 10.1007/978-981-13-0484-2_8 30030794

